# Cost-effectiveness of nivolumab in patients with advanced renal cell carcinoma treated in the United States

**DOI:** 10.1186/s40164-018-0095-8

**Published:** 2018-02-09

**Authors:** Charles McCrea, Sukhvinder Johal, Shuo Yang, Justin Doan

**Affiliations:** 1 Health Economic Modelling Unit, PAREXEL Access Consulting, Evergreen Building North, 160 Euston Road, London, NW1 2DX UK; 2 Bristol-Myers Squibb, Princeton, NJ USA

**Keywords:** Cost-effectiveness, Everolimus, Nivolumab, Renal cell carcinoma, Survival model

## Abstract

**Background:**

We evaluated the cost-effectiveness of nivolumab versus everolimus in patients with advanced renal cell carcinoma (RCC) from a US payer perspective.

**Methods:**

A partitioned survival model consisting of three health states, progression-free survival (PFS), progressive disease, and death, was developed to evaluate the cost-effectiveness of intravenous nivolumab versus oral everolimus over a lifetime. The proportion of patients in each state was calculated based on parametric distributions fitted to PFS and overall survival (OS) data from CheckMate 025 (N = 821), a large randomized phase 3 trial of nivolumab versus everolimus for advanced RCC. Health state utility data were derived from CheckMate 025 EQ-5D data. Scenario analyses and deterministic and probabilistic sensitivity analyses assessed the impact of uncertainty in model inputs on outcomes.

**Results:**

Over a 25-year lifetime horizon, treatment with nivolumab resulted in a gain of 0.64 quality-adjusted life-years (QALYs) versus everolimus. Nivolumab had greater total costs versus everolimus ($US197,089 vs. $US163,902), mainly due to higher acquisition costs. The incremental cost-utility ratio (ICUR), a measure of incremental costs divided by incremental QALYs, was $US51,714 per QALY gained for nivolumab versus everolimus, and an incremental cost-effectiveness ratio was $US44,576 per life-year gained for nivolumab versus everolimus. In sensitivity analyses, average body weight had the greatest impact on the ICUR for nivolumab versus everolimus (base case $US51,714; range $US8863–$US94,566). At a $US150,000 willingness-to-pay (WTP) threshold, nivolumab had a 91.7% probability of being cost-effective versus everolimus.

**Conclusions:**

In the United States, at a WTP threshold of $US150,000 per QALY, nivolumab was found to be cost-effective. Key drivers of cost-effectiveness were survival inputs for OS and the average weight of patients; the latter directly affects nivolumab drug acquisition cost.

**Electronic supplementary material:**

The online version of this article (10.1186/s40164-018-0095-8) contains supplementary material, which is available to authorized users.

## Background

In the United States, kidney cancer is the sixth most common cancer in men and tenth most common cancer in women [1]. In 2017, there were ~ 64,000 new cases and ~ 14,500 deaths as a result of kidney cancer [1]. Renal cell carcinoma (RCC) accounts for ~ 80% of all kidney cancers [2]. In addition to low survival rates for advanced disease [3], there is also a substantial economic burden due to kidney cancer in the United States [4].

Nivolumab is a first-in-class programmed death-1 immune checkpoint inhibitor approved for use in patients with advanced RCC who have received prior antiangiogenic therapy. Approval was based on results from CheckMate 025, a randomized phase 3 trial of nivolumab 3 mg/kg every 2 weeks (N = 410) versus everolimus 10 mg once daily (N = 411) in patients with previously treated advanced RCC [5]. The study met its primary endpoint of overall survival (OS), achieving a median OS of 25.0 months with nivolumab versus 19.6 months with everolimus [5]. Nivolumab was associated with a significantly improved adverse event (AE) profile. Grade 3–4 treatment-related AEs were 19% for nivolumab versus 37% for everolimus [5]. Longer-term survival data are available for a phase 1 (CA209-003) study of nivolumab, which demonstrated a median OS of 22.4 months and a 5-year OS rate of 34% (minimum follow-up, 50.5 months) in patients with advanced RCC [6].

With the increased use of nivolumab as a standard of care in second-line RCC, there is a need to inform US payers on the value of nivolumab. Although cost-effectiveness analyses have been conducted for nivolumab in melanoma and non-small cell lung cancer in a number of countries [7–11], few analyses have been reported for RCC [12, 13].

The objectives of the current study were to evaluate the cost-effectiveness of nivolumab compared with everolimus in patients with advanced RCC from a US payer perspective. To this end, a decision analytical model was developed and validated to estimate the lifetime costs and outcomes of treatments for this patient population.

## Patients and methods

### Health state model structure

Consistent with the approaches adopted in previous economic evaluations and technology appraisals for RCC treatment that were submitted to the Scottish Medicines Consortium and the National Institute for Health and Care Excellence, a 3-state partitioned survival model was developed to evaluate the incremental cost-utility ratio (ICUR) and incremental cost-effectiveness ratio (ICER) of nivolumab versus everolimus. The model comprised 3 key health states representing the primary stages of disease in advanced or metastatic RCC: progression-free (PF), progressive disease (PD), and death. Each state represents a point in the disease pathway at which health-related quality of life (HRQoL) is expected to vary.

The partitioned survival model estimated occupancy of health states using an area-under-the-curve approach from OS and progression-free survival (PFS) parametric curves derived from CheckMate 025. Patient-level data from CheckMate 025 (minimum follow-up, 26 months) were used in the development and validation of the economic model outputs. CheckMate 025 was a large, randomized phase 3 study of nivolumab versus everolimus for previously treated advanced RCC. The time horizon of the analysis was defined as a lifetime (25 years), and a 4-week cycle length (a fixed unit of time by which the model moves forward in time) was selected to accommodate the dosing regimens for nivolumab and everolimus.

### Survival estimates

**Fig. 1:**
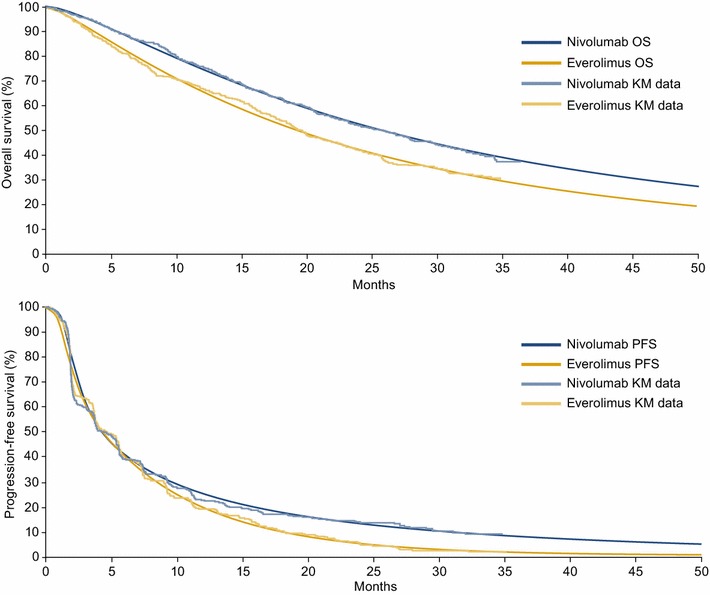
OS (top panel) and PFS (bottom panel) Kaplan–Meier curves with best-fitting parametric curves. KM, Kaplan–Meier; OS, overall survival; PFS, progression-free survival

CheckMate 025 provided data up to a limited time period (minimum follow-up, 26 months; median follow-up, 33.6 months). In order to adopt a lifetime perspective, extrapolation beyond the trial period was required to estimate disease progression and OS over a lifetime. A number of models exist for extrapolating survival. Each model has a different functional form and makes different underlying assumptions on the long-term behavior of survival data, resulting in different survival estimates (and hence ICURs/ICERs). The National Institute for Health and Care Excellence Decision Support Unit has issued guidance on appropriate selection of the extrapolation method, and we followed this selection process to identify appropriate models for nivolumab and everolimus [14]. The fitted curves were assessed in terms of visual inspection of survival curve fit to clinical trial Kaplan–Meier data, inspection of log-cumulative hazard plots (to assess the behavior of the hazards over time), statistical model fit via measures such as Akaike’s information criterion (AIC)/Bayesian information criterion (BIC), and long-term extrapolation estimates versus longer-term data from the phase 1 CA209-003 study (60-month OS time point) of nivolumab for previously treated advanced RCC [6]. Based on the goodness of fit, OS was modeled using the dependent 1-knot spline normal survival function for nivolumab and everolimus (Fig. 1, top panel). PFS was modeled using the dependent 2-knot spline hazard for nivolumab and everolimus, with an adjustment on the shape parameter at gamma 1 and 2 for nivolumab (Fig. 1, bottom panel). Parametric spline models are “structurally flexible” extensions of the standard parametric distributions such as the Weibull, log-normal, and log-logistic functions. They are similar to piecewise modeling as they are flexible mathematical functions defined by piecewise polynomials joined at time points along the curve known as knots. They are particularly useful in cost-utility modeling as they are more flexible models that can better fit the estimated Kaplan–Meier data from clinical trials, particularly when the Kaplan–Meier curves are “unique” and difficult to fit with standard distributions, or when several clinical processes influence the shape of the curve. In the case of nivolumab, spline-based models provided a better fit to the observed data compared with other, more standard parametric models. Other models were tested in a sensitivity analysis.

### Health-related quality of life (utility) estimates

The EuroQol-5 Dimensions (EQ-5D) instrument was used in the CheckMate 025 study to collect HRQoL data by health state. Health state utilities for PF for nivolumab and everolimus were weighted according to the objective response rate estimated for each treatment from CheckMate 025. The odds ratio for treatment response was 5.93 for nivolumab versus everolimus. It was assumed in the economic model that all patients remaining in the PF health state beyond 22 months were considered to have the health state utility of responders. The utility values assigned to each health state were as follows: PF (complete response/partial response), 0.895; PF (stable disease), 0.846; and PD, 0.817. Additional one-off utility decrements were ascribed to patients who experienced AEs.

### Cost estimates

Cost estimates were derived based on the US healthcare system. Only direct medical costs were included in the analysis. These included the cost of drug acquisition, drug administration, monitoring (routine scans), disease management, end-of-life care, and the management of AEs. Patients were treated until progression, although this assumption was tested in a sensitivity analysis. The drug acquisition cost for 10 mg/mL nivolumab was $US2545.15 per vial for 100 mg, and $US13,233.17 for 10 mg of everolimus in 28 tablets [15]. The drug acquisition cost per administration of nivolumab without wastage was $US5451.71. The incidence rates of AEs in the model were derived from CheckMate 025 and included any drug-related grade 3 or higher events with a > 5% incidence in either the nivolumab or everolimus arm. AEs included in the model were hypertriglyceridemia and anemia; the unit cost per event was $US73.06 and $US3237.89, respectively [16, 17]. US-specific inputs related to costs (cost year, 2017) were disease management costs, drug acquisition costs, and unit administration costs ($US139.61 for nivolumab [18]; zero for everolimus because it is orally administered) and routine monitoring costs with the assumption of 1 oncologist visit every 4 weeks ($US79.67) (Additional file 1: Tables S1, S2) [19]. In CheckMate 025, 55% of patients treated with nivolumab and 63% of patients treated with everolimus received subsequent systemic therapy [5]. A subsequent treatment cost was applied as a one-off cost to patients entering the PD health state and was calculated based on therapies received by > 10 patients in CheckMate 025 and an average of 3.65 months’ duration of treatment (Additional file 1: Table S3) [20].

### Scenario and sensitivity analyses

Scenario, deterministic sensitivity, and probabilistic sensitivity analyses were conducted to assess the impact of uncertainty in model inputs on the outcomes. Two scenario analyses were carried out. First, a commonly used standard parametric distribution, a Weibull model, was independently fitted to everolimus and nivolumab OS data. Second, because CheckMate 025 had a significant number of patients treated post progression, the time to treatment discontinuation (TTD) data derived from CheckMate 025 were used in lieu of PFS data in the nivolumab arm to account for differences in PFS and observed TTD which may have resulted due to issues of pseudoprogression in the nivolumab cohort [21]. One-way deterministic sensitivity analyses were conducted with means and plausible ranges for each parameter (Table 1). The following parameters were used: discount rate, body weight, costs, utilities, and clinical data.

**Table 1:** Inputs for deterministic sensitivity analysis

Parameter	Mean (range)
Discount rate—costs, %	3 (0–6)
Discount rate—outcomes, %	3 (0–6)
Average body weight, kg	71.4 (57.1–85.7)
Cost—PF state, $US	65.67 (52.53–78.80)
Cost—PD state, $US	91.61 (73.29–109.93)
Terminal cost, $US	10,713.01 (8570.41–12,855.61)
Administration cost, $US	
Nivolumab	139.61 (111.69–167.53)
Everolimus	Not applicable^a^
Monitoring cost, $US	
Nivolumab	79.67 (63.74–95.60)
Everolimus	79.67 (63.74–95.60)
Utility weight, PF, response	
Nivolumab	0.895 (0.889–0.901)
Everolimus	0.895 (0.889–0.901)
Utility weight, PF, no response	
Nivolumab	0.846 (0.840–0.852)
Everolimus	0.846 (0.840–0.852)
Utility weight, PD	
Nivolumab	0.817 (0.811–0.823)
Everolimus	0.817 (0.811–0.823)

PD, progressive disease health state; PF, progression-free health state

^a^No associated infusion costs, as everolimus is administered orally

Probabilistic sensitivity analysis was used to assess the variation in the model results from the uncertainty around each parameter in the model. Model parameters were sampled from parametric distributions to generate 1000 estimates of the costs and effects in each treatment arm. A gamma distribution was adopted for all costs and resource utilization parameters and a beta distribution was used for the utilities and all probabilities [22]. Drug acquisition costs were exempt from the probabilistic analysis. For the parametric survival curves for PFS and OS, a multivariate normal distribution with correlation between curve parameters was used [22].

## Results

### Base-case results

Over a 25-year horizon, treatment with nivolumab resulted in a quality-adjusted life-year (QALY) gain of 0.64 versus everolimus (Table 2).

**Table 2:** Base-case results for the United States

	Nivolumab	Everolimus	Incremental results
Total costs, $US	197,089	163,902	33,186
Health states, $US			
PF	833	539	
PD	12,596	12,382	
Initial treatment, $US			
Acquisition	138,429	108,859	
Administration	3545	0	
Monitoring	1011	655	
Subsequent treatment, $US			
Acquisition	40,272	40,817	
Administration	92	127	
Monitoring	254	265	
Adverse events, $US	56	257	
Total QALYs (discounted)	2.79	2.15	0.64
PF	0.84	0.54	
PD	1.95	1.62	
AEs	− 0.001	− 0.006	
Total LYG (discounted)	3.36	2.61	0.74
Incremental cost per QALY gained, $US			51,714
Incremental cost per LYG, $US			44,576

AE, adverse event; LYG, life-year gained, PD; progressive disease health state; PF, progression-free health state; QALY, quality-adjusted life-year

Patients treated with nivolumab incurred higher costs, mainly associated with treatment acquisition, in part due to the increased time spent in the PF health state (Table 2). The ICUR of $US51,714 per QALY gained versus everolimus, and an ICER of $US44,576 per life-years gained (LYG) versus everolimus, were estimated (Table 2). AE treatment costs were lower with nivolumab versus everolimus (Table 2).

### Scenario analyses

An investigation into the effect of parametric curve selection on costs, HRQoL, and survival was conducted using Weibull curves fitted separately to nivolumab and everolimus data. The incremental cost per QALY for nivolumab versus everolimus was $US80,439, and the incremental cost per LYG was $US71,697. Incremental costs for nivolumab versus everolimus were $US31,457 and incremental QALYs were 0.39 (Table 3). In a second scenario analysis, a TTD curve was used as proxy for PFS in the nivolumab arm only. When both costs and QALYs were calculated using TTD instead of PFS for nivolumab, the incremental cost per QALY for nivolumab versus everolimus was $US99,574 and the incremental cost per LYG was $US87,391. Incremental costs were $US65,062 and incremental QALYs were 0.65 (Table 3).

**Table 3:** Scenario analyses for nivolumab vs everolimus

Nivolumab vs everolimus	Independent Weibull OS curves	Dependent log-logistic OS curve	Independent 2-knot spline hazard curves fitted to nivolumab and everolimus PFS data	Nivolumab TTD curve is used as a proxy for PFS	Ascribed doses of nivolumab
Incremental cost per QALY gained, $US	80,439	49,827	53,273	99,574	55,591
Incremental cost per LYG, $US	71,697	42,866	45,963	87,391	47,917
Incremental costs, $US	31,457	33,138	34,219	65,062	35,674
Incremental QALYs	0.39	0.67	0.64	0.65	0.64
Median OS nivolumab/everolimus, months	26.3/20.4	26.0/19.1	26.0/19.4	26.0/19.4	26.0/19.4
Mean OS nivolumab/everolimus, months	34.0/28.3	50.9/39.5	45.5/34.8	45.5/34.8	45.5/34.8

LYG, life-year gained; OS, overall survival; PFS, progression-free survival; QALY, quality-adjusted life-year; TTD, time to treatment discontinuation

### Deterministic and probabilistic sensitivity analyses

**Fig. 2:**
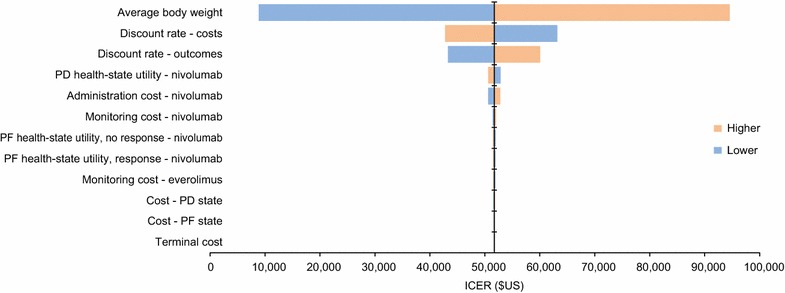
Deterministic sensitivity analysis. Orange bars represent the upper bound of each parameter varied. Blue bars represent the lower bound of each parameter varied. ICER, incremental cost-effectiveness ratio; PD, progressive disease; PF, progression-free

The average body weight had the greatest impact on the ICUR for nivolumab versus everolimus (base case $US51,714; range $US8863–$US94,566; Fig. 2). The ICUR was also sensitive to the discount rate for costs and outcomes (range $US42,771–$US63,179 and $US43,288–$US60,077, respectively). The ICUR did not change substantially across the other parameters tested.

**Fig. 3:**
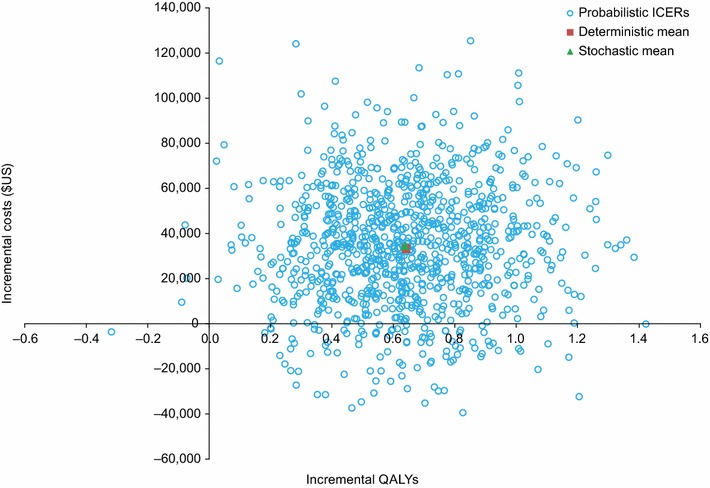
Cost-effectiveness plane (nivolumab vs. everolimus). ICER, incremental cost-effectiveness ratio; QALY, quality-adjusted life-year

Probabilistic sensitivity analysis was conducted to assess the variation in the model results from the uncertainty around each parameter tested. The cost-effectiveness scatterplot for nivolumab versus everolimus is shown in Fig. 3.

**Fig. 4:**
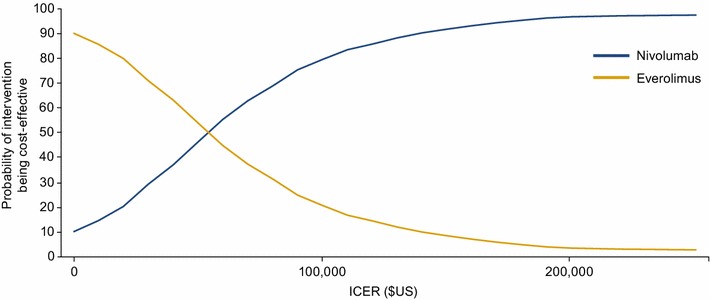
Pair-wise cost-effectiveness acceptability curve (nivolumab vs. everolimus). ICER, incremental cost-effectiveness ratio

At a willingness-to-pay threshold of $US150,000, nivolumab had a 91.7% probability of being cost-effective compared with everolimus alone in the base-case setting. At a willingness-to-pay threshold of $US200,000, nivolumab had a 96.7% probability of being cost-effective compared with everolimus (Fig. 4).

The best-fitting parametric survival models were validated against observed data from CheckMate 025 (minimum follow-up, 26 months) and 60-month survival rates derived from the phase 1 CA209-003 study [5, 6]. Parametric survival models match the Kaplan–Meier data from the clinical trials closely (Table 4).

**Table 4:** Validation of parametric survival models for OS

Data source	OS curve	Proportion in OS (%)										Median OS, month	Mean OS, month
		6 months	1 year	2 years	3 years	4 years	5 years	10 years	15 years	20 years	25 years		
Dependent 1-knot spline normal (base-case analysis)	NIVO	89.9	75.0	53.0	38.5	29.0	22.4	8.3	3.9	2.2	1.3	26.0	45.5
	EVE	84.4	66.0	42.6	29.0	20.7	15.4	4.9	2.2	1.1	0.6	19.4	34.8
Independent Weibull curves	NIVO	90.0	76.5	53.9	36.6	24.1	15.6	1.4	0.1	< 0.01	< 0.001	26.3	34.0
	EVE	84.3	67.6	43.9	28.0	17.6	11.0	1.0	0.1	< 0.01	< 0.001	20.4	28.3
Dependent log-logistic curve	NIVO	89.7	74.8	53.0	39.0	29.9	23.8	10.6	6.3	4.3	2.9	26.0	50.9
	EVE	85.0	65.9	42.3	29.3	21.7	16.9	7.2	4.2	2.8	1.9	19.1	39.5
CheckMate 025	NIVO	89.2	76.0	–	–	–	–	–	–	–	–	25.0	–
	EVE	81.2	66.7	–	–	–	–	–	–	–	–	19.55	–
CA209-003	NIVO	82.4	70.6	48.1	41.2	37.8	34.3	–	–	–	–	22.4^a^	–
	EVE	–	–	–	–	–	–	–	–	–	–	–	–

The median OS for 1 mg/kg dosing group was 29.3 months; for 10 mg/kg dosing group, the median OS was 18.8 months

EVE, everolimus; NIVO, nivolumab; OS, overall survival

^a^Both 1 and 10 mg/kg

Based on the observed 60-month OS rate with nivolumab in CA209-003 (34.3%) [6], the 60-month OS rate estimated using the base-case scenario (dependent 1-knot spline normal) may underestimate survival (22.4%) (Table 4). Scenarios using the dependent log-logistic (23.8%) and independent Weibull distributions may also underestimate survival (15.6 and 11.0% for nivolumab and everolimus, respectively) compared with the observed 60-month OS rate in CA209-003 (Table 4).

## Discussion

The results of the economic evaluation described here indicate that at a cost of $US2545.15 per 100-mg vial, nivolumab has an ICUR of $US51,714 per QALY gained over everolimus and an ICER of $US44,576 per LYG over everolimus. At a willingness-to-pay threshold of $US150,000, nivolumab had a 91.7% probability of being cost-effective compared with everolimus. The key drivers of cost-effectiveness were the survival inputs for OS and the average weight of patients, the latter having a direct effect on the drug acquisition cost of nivolumab. Validation with long-term RCC survival data in the real-world setting is limited due to lack of data beyond 5 years. Although the dependent 1-knot spline normal is the best-fitting curve for OS observed in CheckMate 025 based on AIC and BIC criteria, the long-term projections indicate that the dependent log-logistic curves may provide a better fit, but are still a conservative assumption compared with CA209-003 5-year data. However, it should be noted that although CA209-003 provides long-term survival estimates, the trial population included previously treated patients who received a range of doses of nivolumab [6], which may explain some of the differences in long-term survival versus CheckMate 025 projections.

For this economic evaluation, the wholesale acquisition cost of nivolumab was used. However, the actual price of nivolumab in the United States may be lower, according to price negotiations with pharmacies. Therefore, the results presented may be considered conservative.

The cost-effectiveness of nivolumab was explored in Sweden using a 3-state partitioned survival model [12]. Nivolumab was found to be cost-effective at established thresholds when accounting for treatment costs that continued through progression [12]. In contrast, Wan et al. concluded that nivolumab was not cost-effective from US and Chinese healthcare system perspectives [13]. Results from the current analysis differed, likely because there were some key differences between approaches, with the current analysis utilizing individual patient-level data and employing a robust methodology to select appropriate distributions to model OS that underwent further validation using long-term CA209-003 study data. Wan et al. used a Weibull distribution to model OS independently for the nivolumab and everolimus arms, with the rationale that it is widely used in cancer survival analyses. There did not appear to be any consideration of goodness of fit against the trial data compared with other parametric models or any attempt to validate longer-term OS predictions. As part of a scenario analysis, we used independent Weibull distributions for OS. The Weibull distribution resulted in a lower mean OS for nivolumab and everolimus and did not reflect the long-term survival benefit of nivolumab observed in CA209-003, providing much lower 60-month survival estimates versus the dependent 1-knot spline normal survival function used in the base-case analysis. Wan et al. also used a less mature dataset from CheckMate 025, which may have resulted in a higher degree of uncertainty in longer-term OS extrapolations.

There are several limitations to our analysis that deserve consideration. The economic model utilizes PFS as a proxy for treatment duration. However, in the nivolumab arm, there were differences in the PFS and TTD curves, indicating that a proportion of patients were treated post progression. Some of those patients may have had pseudoprogression, an observed phenomenon caused by the unconventional immune-related response that gives the initial appearance of progression [23]. Thus, the costs and benefits of nivolumab treatment may be underestimated in the base-case analysis. The ICUR increased substantially when the TTD curve was used to calculate drug acquisition, drug administration, and monitoring costs for nivolumab; however, it is important to consider that the quality-of-life benefits associated with increased treatment duration on nivolumab were not captured in this scenario. In addition, only AEs with > 5% incidence at grade 3 or higher severity were included. This could underestimate the impact of AEs in the model because nivolumab has a more favorable tolerability profile than everolimus. However, it should be noted that AEs contributed to < 1% of the incremental costs of nivolumab versus everolimus. Finally, long-term extrapolation of OS curves from short-term clinical trials is always subject to uncertainty, and hence should be validated against long-term data from other sources. However, long-term validation proved difficult due to a lack of real-world evidence for advanced RCC. Where data were available, the model was validated against long-term survival data and different parametric distributions for OS were tested in scenario analyses.

The standard dose of nivolumab for RCC was recently modified from 3 mg/kg every 2 weeks to 240 mg every 2 weeks [24] based on population pharmacokinetics and dose/exposure–response analyses. A dose and schedule of 480 mg every 4 weeks is currently being explored, which may improve the cost-effectiveness of nivolumab in this patient population because fewer office visits would be needed for drug administration.

The results of the economic evaluation need to be considered in the context of the high unmet need/poor survival within previously treated advanced RCC [2]. The 5-year survival for metastatic RCC is 11.7% [3]. There is also a substantial cost of illness for metastatic RCC, with patients experiencing high out-of-pocket costs [25–27]. Effective strategies to reduce the prevalence and progression of RCC have the potential to considerably reduce the economic burden. Studies on treatment sequence and schedule that may help to maximize benefit and optimize cost-effectiveness should be explored. Clinical data of nivolumab in previously treated patients with advanced RCC presents a compelling case that nivolumab represents a “step-change” in disease management with improved OS, favorable tolerability, and improved quality of life compared with everolimus [5, 28].

At this juncture, physicians and payers alike are making second-line treatment decisions for patients with RCC with nivolumab and cabozantinib in mind. This analysis may be extended to an indirect comparison of cost-effectiveness of these two agents.

## Conclusions

In addition to the known survival benefit, improved HRQoL, and favorable tolerability profile of nivolumab compared with everolimus, we also found nivolumab to be cost-effective for patients with advanced RCC at a $US150,000 per QALY threshold in the United States, even when taking into account the higher costs of initial treatment acquisition, administration, and monitoring. The model structure developed here may serve as a template for assessing cost-effectiveness of nivolumab in other countries and for comparison with other relevant treatments for second-line RCC.

## Additional file

**Additional file 1.** Additional tables.

## Abbreviations

AE: adverse event
AIC: Akaike’s information criterion
BIC: Bayesian information criterion
EQ-5D: EuroQol-5 Dimensions
HRQoL: health-related quality of life
ICER: incremental cost-effectiveness ratio
ICUR: incremental cost-utility ratio
LYG: life-years gained
OS: overall survival
PD: progressive disease
PF: progression-free
PFS: progression-free survival
QALY: quality-adjusted life-year
RCC: renal cell carcinoma
TTD: time to treatment discontinuation
