# Supplementary material for: Cost-effectiveness of nivolumab in patients with advanced renal cell carcinoma treated in the United States
Source: Exp Hematol Oncol. 2018 Feb 9;7:4. doi: 10.1186/s40164-018-0095-8 (PMC5810189; doi:10.1186/s40164-018-0095-8)
Supplement: Supplementary file 1 — Additional file 1. Additional tables. [file 40164_2018_95_MOESM1_ESM.pdf]

Additional file 1

Table S1 Disease management costs (PF and PD health states) and end-of-life/terminal care costs

Resource name	No. required per 4 weeks	Unit cost ($US)	Reference
**PF**			
Office visit	0.104	79.67	CMS Physician Fee Schedule. HCPCS code: 99214 (office visit, established patient, facility price), National payment amount
Community nurse visit	0	25.84	CMS Physician Fee Schedule. HCPCS code: 99212 (office visit, established patient, facility price), National payment amount
CT scan	0.028	467.99	CMS Physician Fee Schedule. HCPCS code: 74160 (CT abdomen w/ dye, facility price, all modifiers), National payment amount
MRI	0.036	920.90	CMS Physician Fee Schedule. HCPCS code: 74182 (MRI abdomen w/ dye, facility price, all modifiers), National payment amount
Blood test, CBC	0.081	8.87	CMS Physician Fee Schedule: 85004, Medicare Laboratory Fee Schedule (2017)
Comprehensive metabolic panel	0.756	11.60	CMS Laboratory Fee Schedule: 80048, Medicare Laboratory Fee Schedule (2017)
Morphine sulphate, oral	0.019	0.89	WAC, Medi-Span; 15 mg every 4 hours
Morphine sulphate, IV drug acquisition	0	6.76	WAC, Medi-Span (assumed 10 mg per day dosage)
Morphine sulphate, IV administration	0.027	58.14	CMS Physician Fee Schedule. HCPCS code: 96376 (injection, facility price), National payment amount
Oxycodone HCl, oral	0.051	1.01	WAC, Medi-Span; 60 mg every 4 hours
**PD**			
Office visit	0.188	79.67	CMS Physician Fee Schedule. HCPCS code: 99214 (office visit, established patient, facility price), National payment amount
Community nurse visit	0	25.84	CMS Physician Fee Schedule. HCPCS code: 99212 (office visit, established patient, facility price), National payment amount
CT scan	0.044	467.99	CMS Physician Fee Schedule. HCPCS code: 74160 (CT abdomen w/ dye, facility price, all modifiers), National payment amount
MRI	0.047	920.90	Centers for Medicare & Medicaid Service - Physician Fee Schedule. HCPCS code: 74182 (MRI abdomen w/ dye, facility price, all modifiers), National payment amount
Blood test, CBC	0.079	8.87	CMS Physician Fee Schedule: 85004, Medicare Laboratory Fee Schedule (2017)
Comprehensive metabolic panel	0.736	11.60	CMS Laboratory Fee Schedule: 80048, Medicare Laboratory Fee Schedule (2017)
Morphine sulphate, oral	0.033	0.89	WAC, Medi-Span; 15 mg every 4 hours

Resource name	No. required per 4 weeks	Unit cost ($US)	Reference
Morphine sulphate, IV drug acquisition	0.001	6.76	WAC, Medi-Span (assumed 10 mg per day dosage)
Morphine sulphate, IV administration	0.058	58.14	CMS Physician Fee Schedule. HCPCS code: 96376 (injection, facility price), National payment amount
Oxycodone HCl, oral	0.11	1.01	WAC, Medi-Span; 60 mg every 4 hours
**End-of-life/terminal care costs**			
Resource Name	No. Required	Unit Cost ($US)	Reference
End–of-life care	1	10,713.01	Cost initially sourced from Perrin et al. and inflated from 2014 to 2017. Values using a “Medical care” CPI in Table 25, page 82 of 121 [https://www.bls.gov/cpi/cpid1705.pdf]

CBC, complete blood count; CMS, US Centers for Medicare and Medicaid Services; CPI, consumer price index; IV, intravenous; PD, progressive disease health state; PF, progression-free disease health state; WAC, wholesale acquisition cost

Table S2 Drug acquisition costs used in the base-case model

Treatment	Formulation per vial/cap	Vial size or tablets per pack	Unit cost per vial or pack 2015 ($US)	Reference
**Nivolumab**	10 mg/mL	10 mL	2545.15	Medi-Span WAC price
**Nivolumab**	10 mg/mL	4 mL	1018.06	Medi-Span WAC price
**Everolimus**	10 mg	28	13,233.17	Medi-Span WAC price
**Axitinib**	5 mg	60	13,680.23	Medi-Span WAC price
**Pazopanib**	200 mg	120	11,018.87	Medi-Span WAC price
**Sunitinib**	50 mg	28	16,208.76	Medi-Span WAC price
**Temsirolimus**	25 mg/mL	1 mL	1718.05	Medi-Span WAC price
**Bevacizumab**	25 mg/mL	16 mL	3034.16	Medi-Span WAC price
**Sorafenib**	200 mg	120	16,479.43	Medi-Span WAC price
**Cabozantinib**	20 mg	84	14,788.84	Medi-Span WAC price

WAC, wholesale acquisition cost

Table S3 Subsequent treatments used in base-case model (treatment duration based on the Global Oncologic Learnings for Dovitinib in Renal Cell Carcinoma Trial[a])

Subsequent treatment	Cost of treatment[b] ($US)	Nivolumab arm	Everolimus arm
**Nivolumab**	44,685.99	0%	0%
**Everolimus**	52,821.96	48%	9%
**Axitinib**	50,977.14	45%	60%
**Pazopanib**	41,121.51	17%	26%
**Sunitinib**	48,419.59	13%	14%
**Temsirolimus**	29,799.00	5%	5%
**Bevacizumab**	44,402.41	6%	9%
**Sorafenib**	61,343.22	12%	15%
**Best supportive care**	316.11	0%	0%
**Cabozantinib**	58,994.46	8%	3%

[a] Reference: Lancet Oncol. 2014;15:286–96.

[b] For a treatment duration of 3.65 months. Costs include drug acquisition, drug administration, and monitoring
